# Gulllain-Barre Syndrome After Trivalent Influenza Vaccination in Adults

**DOI:** 10.3389/fneur.2019.00768

**Published:** 2019-07-24

**Authors:** Kuo-Hsuan Chang, Rong-Kuo Lyu, Wan-Ting Lin, Yu-Tung Huang, Huang-Shen Lin, Shang-Hung Chang

**Affiliations:** ^1^Department of Neurology, Chang Gung Memorial Hospital, Taoyuan, Taiwan; ^2^College of Medicine, Chang Gung University, Taoyuan, Taiwan; ^3^Center for Big Data Analytics and Statistics, Chang Gung Memorial Hospital, Taoyuan, Taiwan; ^4^Division of Infectious Diseases, Chang Gung Memorial Hospital, Chiayi, Taiwan; ^5^Division of Cardiology, Chang Gung Memorial Hospital, Taoyuan, Taiwan; ^6^Graduate Institute of Nursing, Chang Gung University of Science and Technology, Taoyuan, Taiwan

**Keywords:** Guillain–Barre syndrome, influenza, endemic flu, vaccination, polyneuropathy

## Abstract

Lines of evidence suggest trivalent influenza vaccination may be associated with Guillain–Barre syndrome (GBS), an immune-mediated acute inflammatory neuropathy. On the other hand, this vaccination protects against influenza infection, which has been demonstrated as a trigger of GBS. To clarify the net effect of trivalent influenza vaccines on GBS, we conducted a retrospective nationwide nested case–control study using the database of the Taiwan National Health Insurance program. We identified 182 hospitalized patients with GBS aged ≥50 years from 2007 to 2015 as the cases, and 910 hospitalized patients, matched by gender, age, date of hospitalization, comorbidities, and medications, as the control subjects. Nearby and remote exposures of vaccination were defined as subjects who had received trivalent influenza vaccine 42 (nearby exposure) and 90 days (remote exposure) before the date of hospitalization, respectively. We found 7 (3.85%) GBS patients and 26 (2.86%) matched control subjects who demonstrated nearby exposures of influenza vaccine (odds ratio: 1.46, 95% confidence interval: 0.56–3.78). Seventeen (9.34%) GBS patients were exposed to influenza vaccines remotely, while the number of remote exposure of influenza vaccines in matched control subjects was 72 (7.91%, odds ratio: 1.26, 95% confidence interval: 0.67–2.38). These results do not support an association between trivalent influenza vaccine and GBS among the patients aged ≥50 years.

## Introduction

Guillain–Barre syndrome (GBS) is an acquired inflammatory peripheral neuropathy characterized by acute limb weakness and arreflexia; present incidence increases with age, with an excess in males (1). Up to 30% of GBS patients develop respiratory failure and require mechanical ventilation (2). The pathogenesis of GBS is thought to be mainly immune-mediated (3). This immune response, triggered by preceding gastrointestinal or upper respiratory tract infections, including influenza (1, 4–8), may generate antibodies that cross-react with gangliosides at myelin, resulting in demyelination and damage to the peripheral nerves (9). On the other hand, the possible association between influenza vaccines and GBS has been a matter of particular concerns (10–23). For example, A/New Jersey/76 “swine” influenza vaccination during 1976–1977 was associated with a seven-fold excess risk of GBS in the subsequent 6-week period in the United States (12). Similarly, an increased risk of GBS after influenza A (H1N1) 2009 monovalent inactivated vaccine was detected (22). In contrast, a study in England found no association between any vaccination and subsequent GBS risk (18).

The most widely used seasonal influenza vaccine is the trivalent inactivated vaccine (24), which protects against two influenza A viruses and one influenza B virus (25). Although this vaccine has a good safe record, its association with GBS has not be excluded. Lines of early studies found increase risk of GBS in the patients receiving trivalent influenza vaccination (11, 15, 17, 21), whereas other studies showed neutral (10, 13, 14, 18–20, 23) or even reduction of GBS risk following vaccination (16). All of these studies are focused on the GBS events within 6–8-week post-vaccination periods. The remote net effect of vaccination is still unknown. Here, we aimed to describe the recent and remote associations between GBS and prior status of trivalent influenza vaccination by a nationwide nested case–control study.

## Subjects and Methods

### Ethics Statement

This study was approved by the Institutional Review Board (IRB no. 201800205B1) of Chang Gung Memorial Hospital, Taiwan. Since all identifying personal information was stripped from the secondary files before analysis, the review board waived the requirement for written informed consent from the patients involved.

### Database

The National Health Insurance (NHI) program provides compulsory universal health insurance in Taiwan, since 1995. The National Health Insurance Research Databases (NHIRD) are claim-based electronic records from the NHI program. In these databases, medical information of disease diagnosis, prescription drugs, procedures, and surgery incurred during a hospitalization or at an outpatient visit are documented. The study data were obtained from the NHIRD between 2007 and 2015 released by the Health and Welfare Data Science Center, Department of Statistics, Ministry of Health and Welfare in Taiwan.

### Study Population

In Taiwan, seasonal trivalent influenza vaccinations were freely offered to the subjects older than 50 years in winter, mostly between October and February, upon request by the Taiwan Centers for Disease Control (TwCDC). Therefore, we recruited all subjects with a recorded age ≥50 in the data set from October 1 to April 30 between 2007 and 2015. The diagnosis of GBS was according to the *International Classification of Diseases, Ninth Revision, Clinical Modification* (*ICD-9-CM*) code 357.0. To ensure diagnostic validity and patient homogeneity, only patients with billing records of hospitalization and plasmapheresis were recruited in the GBS group.

### Exposure Definition

To investigate recent (within 42 days after vaccination) and remote (within 90 days after vaccination) associations between vaccination and GBS, we retrospectively reviewed the vaccination history before the onset of GBS. Because of the policy of free influenza vaccination starting from October 1 each year (closed upon run out, in general on January or February) setting by TwCDC. Considering 42 and/or 90 days of trace back in this study, we only recruited target subjects with a record from October 1 to April 30 between 2007 and 2015. The vaccine exposures were defined by billing records of all inactivated, split, not-adjuvanted, trivalent, and seasonal influenza vaccines available in Taiwan, including Agrippal (Novartis Vaccine), Vaxigrip (Pasteur Merieux Connaught), Fluarix (Glaxo SmithKline), and AdimFlu-S (Adimmune).

### Potential Confounding Variables and Matching Control Subjects

Patient demographics included age, gender, and socioeconomic factors (residence, income level, and occupation) were identified as covariates. We considered comorbidities [acute respiratory infection, pneumonia, influenza, urinary tract infection, intestinal infection, human immunocompromised virus (HIV) infection, hematological neoplasms, diabetes mellitus, herpes infection, chronic kidney disease, hepatic diseases, viral hepatitis, and connective tissue diseases] and medications [antibiotics, anti-HIV drugs, chemotherapeutic agents, angiotensin-converting enzyme inhibitors (ACEI), angiotensin II receptor blockers, aliskiren, and antidepressants] believed or reported to affect the risk of GBS as potential confounders (26–28). The comorbidities were identified by *ICD-9-CM* codes (Table 1) and only counted while co-occurring at the hospitalization or emerging more than two times within 90 days prior to the hospitalization. The medications within 90 days prior to the hospitalization were recorded. The control subjects were completely matched to the patient with GBS by comorbidities and medications with a ratio of 1: 5.

**Table 1:** Comorbidities for evaluation of association between GBS and trivalent influenza vaccine.

**Comorbidities**	***ICD-9* code**
Acute respiratory infection	460–466
Pneumonia and influenza	480–488
Urinary tract infection	599.0
Intestinal infection	008–009
HIV infection	042
Hematological neoplasms	200–209
DM	250
Flu	487
Herpes infection	054
Chronic kidney disease	585
Liver disorders	573
Viral hepatitis	070
Connective tissue disorders	710, 714, 695.4, 446

### Statistical Analysis

Demographic data are expressed as mean [± standard deviation (SD)] or percentage. In general, differences in proportions were tested with the chi-square test or Fisher's exact test, and differences in continuous variables were tested with Student's *t*-test. Random matching procedures were performed by SAS statistical software and were based on random numbers generated from the uniform distribution. The entire analysis was performed using SAS (SAS Institute), version 9.4.

## Results

Our study included 182 GBS patients and 910 control subjects without GBS with hospitalization. Comparisons of demographic and clinical variables between GBS patients and control subjects are presented in Table 2. Median age at enrolment was 65.19 ± 9.94 years in GBS patients and 65.20 ± 9.91 years in control subjects. The gender distributions (male/female: 62.09%/37.91%) of both groups are identical. GBS patients demonstrated similar income levels and occupations. The comorbidities, including acute respiratory infection, pneumonia, influenza, urinary tract infection, intestinal infection, HIV infection, hematological neoplasms, diabetes mellitus, herpes infection, chronic kidney disease, hepatic diseases, viral hepatitis, and connective tissue diseases controls are completely matched within groups (Table 3). Around three-fourths of GBS patients and control subjects (74.18%) were exposed to antibiotics before or at the hospitalization, while 52.75% of subjects received renin–angiotensin system blockades. No enrolled subjected received treatment with tumor necrosis factor-α (TNF-α) inhibitor and anti-HIV medications.

**Table 2:** Demographic features of patients with GBS and control subjects.

**Variables**	GBS patients (*n* = 182)	Control subjects (*n* = 910)	***p* value**
	**No. (%)**	**No. (%)**	
Age, year, mean (SD)	65.19 (9.94)	65.20 (9.91)	0.99
Gender			>0.99
Male	113 (62.09)	565 (62.09)	
Female	69 (37.91)	345 (37.91)	
Place of residence			0.27
Urban	104 (57.14)	493 (54.18)	
Suburban	60 (32.97)	286 (31.43)	
Rural	18 (9.89)	131 (14.40)	
Income levels			0.83
Quintile 1 (Lowest)	44 (24.18)	217 (23.85)	
Quintile 2	36 (19.78)	214 (23.52)	
Quintile 3	25 (13.74)	121 (13.30)	
Quintile 4	36 (19.78)	177 (19.45)	
Quintile 5 (Highest)	41 (22.53)	181 (19.89)	
Occupation			0.67
Dependents of the insured individuals	62 (34.07)	289 (31.76)	
Civil servants, teachers, military personnel, and veterans	14 (7.69)	49 (5.38)	
Non-manual workers and professionals	16 (8.79)	90 (9.89)	
Manual workers	60 (32.97)	311 (34.18)	
Others	30 (16.48)	171 (18.79)	

**Table 3:** Comorbidities and medications of patients with GBS and control subjects.

**Variables**	GBS patients (*n* = 182)	Control subjects (*n* = 910)	***p* value**
	**No. (%)**	**No. (%)**	
**Comorbidities**			
Acute respiratory infection	150 (82.42)	750 (82.42)	>0.99
Pneumonia and influenza	38 (20.88)	190 (20.88)	>0.99
Urinary tract infection	46 (25.27)	230 (25.27)	>0.99
Intestinal infection	<5	15 (1.65)	>0.99
HIV infection	0 (0)	0 (0)	–
Hematological neoplasms	0 (0)	0 (0)	–
Diabetes mellitus	59 (32.42)	295 (32.42)	>0.99
Influenza	6 (3.30)	30 (3.30)	>0.99
Herpes infection	0 (0)	0 (0)	–
Chronic kidney disease	7 (3.85)	35 (3.85)	>0.99
Liver disorders	<5	5 (0.55)	> 0.99
Viral hepatitis	7 (3.85)	35 (3.85)	>0.99
Connective tissue disorders	<5	5 (0.55)	>0.99
**Drug use**			
Antibiotics	135 (74.18)	675 (74.18)	>0.99
Anti-HIV	0 (0)	0 (0)	–
Chemotherapy	<5	20 (2.20)	>0.99
ACEI or ARB or aliskiren	96 (52.75)	480 (52.75)	>0.99
SSRI	<5	15 (1.65)	>0.99
Other antidepressants	12 (6.59)	60 (6.59)	>0.99
TNF-α inhibitor	0 (0)	0 (0)	–

*ARB, angiotensin receptor blocker; SSRI, selective serotonin reuptake inhibitor*.

The percentage of trivalent influenza vaccine within 90 days prior to hospitalization in GBS patients was 9.34%, which did not significantly differ from those in control subjects (7.91%, odds ratio: 1.26, *P* = 0.47, Table 4). Similar proportions of GBS patients (3.85%) and control subjects (2.86%) received seasonal trivalent influenza vaccine within 42 days prior to hospitalization (odds ratio: 1.46, *P* = 0.44).

**Table 4:** Odds ratio of history of trivalent influenza vaccination on GBS patients and control subjects (using conditional logistic regression).

	GBS patients (*n* = 182)	Control subjects (*n* = 910)	Odds ratio (95% CI)	***p* value**
	**No. (%)**	**No. (%)**		
Flu vaccine within 90 days	17 (9.34)	72 (7.91)	1.26 (0.67–2.38)	0.47
Flu vaccine within 42 days	7 (3.85)	26 (2.86)	1.46 (0.56–3.78)	0.44

*CI, confidence interval*.

## Discussion

This population-based nested case–control study showed absent association between GBS and preceding trivalent influenza vaccination within 42 and 90 days by estimating the number of vaccination in GBS patients. Our approach enrolls a large number of GBS patients, in contrast to the self-controlled series, which only detect a limited number of GBS patients following vaccination (11, 13, 15, 17, 21, 23). To eliminate the inherent risk for GBS regardless of vaccination, we also control all available confounding factors that are attributes of the cases and that do not change appreciably during the study period, such as age, gender, comorbidities, and medications. Therefore, our study provide minimally biased information about the safety of trivalent influenza vaccines by using a real-world nationwide healthcare database.

The potential association between GBS and flu vaccine might vary geographically or ethnically. From 1990 to 2003, the American Vaccine Adverse Event Reporting System received 501 reports of GBS following influenza vaccine, and the most common interval from immunization to the onset of neuropathy was 2 weeks (29). A meta-analysis in 2015 revealed a marginally statistically significant associations between trivalent influenza vaccines and post-vaccination GBS within 6 weeks. However, the subgroup analysis revealed inconsistent results in different geographical location (30). This association is strong and significant in the United States and Canada, whereas European studies do not recapitulate this association (30). In China, no potentially vaccine-associated cases of the GBS were identified among 95,244 cases receiving pandemic monovalent influenza A vaccine (31). Our study is the first nationwide population-based case–control study to evaluate association of GBS with prior exposure of trivalent influenza vaccine. Our results may support the geographical or ethnic difference of the development of GBS following vaccination.

The relationship between vaccines and GBS might be bidirectional. On the one hand, vaccines may trigger autoimmunity and develop autoimmunity (32). On the other hand, influenza infection can act as triggers for GBS. A study of UK database in 1990–2001 showed an increased risk of GBS within 2 months of an influenza-like illness or acute respiratory infection, whereas this increase in risk was not seen after vaccination (33). Moreover, vaccination may reduce the risk of adverse outcomes. This benefits may be particularly important in older adults, who are at high risk for complications from influenza. Vaccination in community-dwelling adults aged ≥50 years demonstrate significantly protective effect against influenza hospitalization (34), supporting current recommendation for annual influenza vaccination among older adults. Our results of absent association between trivalent influenza vaccine and GBS further enhance the benefit of influenza vaccination prevailing over the potential risk of GBS.

GBS is believed to be an immune disorder resulting from the generation of autoimmune antibodies against gangliosides on peripheral nerves, such as anti-GM1, anti-GM2, or anti-GD1b antibodies. Monovalent and bivalent vaccines against swine flu in 1976 can induce anti-GM1 antibodies after inoculation into mice (35). Anti-GM2 antibodies can be detected in a patient who developed acute disseminated encephalomyelitis and GBS associated with the monovalent influenza vaccine against influenza A as well (36). Anti-GD1b antibodies have been reported in a GBS patient exposed to influenza A infection (37). Assays of these anti-ganglioside antibodies in the patients who previously had GBS may be important before receiving influenza vaccination.

Our results provide reassurance that the majority of patients with GBS in Taiwan are not associated with seasonal trivalent influenza vaccination. Cautious interpretation should be taken because the distinct influenza vaccines prepared at different times may be associated with varying risks (12, 14). Our data were obtained from electronic clinical records of practices and may be affected by the general limitations of this source, including potential problems of misdiagnosis, lack of detail about the precise time of onset, lack of standardization, and insufficient verification. The incomplete vaccine coverage may reduce number of vaccine exposure of included patients. The patient recruitment may be limited to severe cases. Some patients with minor symptoms who did not receive plasmapheresis or those receiving self-paid treatment such as intravenous immunoglobulin are not included in this study. Our results refer only to the trivalent vaccines and cannot be generalized to all influenza vaccines. Nevertheless, in view of the generally serious nature of GBS and the relatively specific clinical picture, these problems may have been fewer than usual. The precise evaluation of the potential risk associated with influenza vaccines by more GBS patients would be warranted.

## Data Availability

The datasets generated for this study are available on request to the corresponding author.

## Author Contributions

K-HC, S-HC, and R-KL conceived and designed the experiments. W-TL performed the experiments. W-TL, K-HC, Y-TH, S-HC, and H-SL analyzed the data. K-HC and S-HC wrote the paper.

### Conflict of Interest Statement

All authors are employees of Chang Gung Memorial Hospital and report no financial disclosures.
